# Whole-Tumor ADC Texture Analysis Is Able to Predict Breast Cancer Receptor Status

**DOI:** 10.3390/diagnostics13081414

**Published:** 2023-04-14

**Authors:** Madalina Szep, Roxana Pintican, Bianca Boca, Andra Perja, Magdalena Duma, Diana Feier, Flavia Epure, Bogdan Fetica, Dan Eniu, Andrei Roman, Sorin Marian Dudea, Angelica Chiorean

**Affiliations:** 1 Department of Radiology, “Iuliu Hatieganu” University of Medicine and Pharmacy, 400347 Cluj-Napoca, Romania; madalinaszep@gmail.com (M.S.);; 2 Department of Medical Imaging, “Iuliu Hatieganu” University of Medicine and Pharmacy, 400347 Cluj-Napoca, Romania; 3 Department of Radiology and Medical Imaging, County Clinical Emergency Hospital, 400347 Cluj-Napoca, Romania; 4 Medimages Breast Center, 400462 Cluj-Napoca, Romania; 5 Medical Imaging Department, Medisprof Cancer Center, 400641 Cluj Napoca, Romania; 6 Department of Pathology, “Ion Chiricuţă” Oncology Institute, 400015 Cluj-Napoca, Romania; 7 Department of Surgical Oncology, “Iuliu Hatieganu” University of Medicine and Pharmacy, 400347 Cluj-Napoca, Romania; 8 Department of Radiology, “Ion Chiricuță” Oncology Institute, 400015 Cluj-Napoca, Romania

**Keywords:** ADC, radiomics, prediction, receptor, breast cancer

## Abstract

There are different breast cancer molecular subtypes with differences in incidence, treatment response and outcome. They are roughly divided into estrogen and progesterone receptor (ER and PR) negative and positive cancers. In this retrospective study, we included 185 patients augmented with 25 SMOTE patients and divided them into two groups: the training group consisted of 150 patients and the validation cohort consisted of 60 patients. Tumors were manually delineated and whole-volume tumor segmentation was used to extract first-order radiomic features. The ADC-based radiomics model reached an AUC of 0.81 in the training cohort and was confirmed in the validation set, which yielded an AUC of 0.93, in differentiating ER/PR positive from ER/PR negative status. We also tested a combined model using radiomics data together with ki67% proliferation index and histological grade, and obtained a higher AUC of 0.93, which was also confirmed in the validation group. In conclusion, whole-volume ADC texture analysis is able to predict hormonal status in breast cancer masses.

## 1. Introduction

Nowadays, it is widely accepted that breast cancer is a multifactorial disease driven by genetic changes and traditional classifications based on tumor histology, size, grade and receptor status no longer capture all its characteristics. Gene expression profiling (GEP) revealed four main intrinsic molecular breast cancer subtypes with differences in incidence, treatment response, outcome and prognosis [1,2,3]. However, GEP is expensive and not widely available. Immunohistochemistry (IHC) procedures using protein expression have been employed as a method for subtyping breast cancer in clinical practice [4]. IHC describes surrogate breast cancer subtypes classified based on the presence or absence of estrogen (ER) and progesterone (PR) receptors, ki67% proliferation index and HER2 status. The five surrogate subtypes consist of luminal A and luminal B (HER2 negative and HER2 positive), which are positive for ER and/or PR, HER2 positives, which are negative for ER/PR and triple negative, ER/PR and HER2 negative. The latter use estrogen and progesterone receptor status and ki67% proliferation index to differentiate between the five types of cancers and do not rely on the genetic method. IHC has been widely accepted because it is faster and more cost-effective. However, IHC analysis can be limited, first, as biopsy captures only a small part of the breast tumor, and second, because high variability was reported across different laboratories when reporting receptor status [5,6].

The emerging field of radiomics relies on extracting imaging features that human eyes are not able to assess or quantify. Previous breast cancer studies which used radiomics to predict molecular subtypes were primarily focused on dynamic contrast-enhanced MRI (DCE-MRI) [7,8,9,10,11]. Less is known about radiomic features derived from diffusion-weighted imaging (DWI) with apparent diffusion coefficient (ADC). Furthermore, the results reported for the last method are still inconclusive, with some authors reporting lower ADC values and positive correlations with molecular subtypes, but with modest AUCs of 0.68–0.718 [12,13,14], and other authors reporting no correlation with pathology characteristics [15]. The conflicting results may be due to different acquisition techniques (different b-values), different segmentation techniques (single/multiple region-of-interest (ROI) versus whole-volume ADC) and small sample sizes. Furthermore, due to the recent concerns about the safety of gadolinium-based contrast agents, there is an increasing interest in developing unenhanced MRI techniques that are able to characterize breast cancer masses [16].

Our study aims to assess the diagnostic performance of radiomic features extracted from whole-tumor ADC texture analysis using standardized DWI acquisition in order to predict breast cancer receptor status and molecular subtypes.

## 2. Materials and Methods

### 2.1. Patients

This retrospective study was approved by the Institutional Ethics Committee (NR10/15092022, from 15 September 2022) and the need for informed consent was waived. We included patients with breast cancer (regardless of disease stage) who presented to our clinic (MBC) from January to September 2022. All patients underwent breast MRIs prior to any treatment with a standardized protocol consisting of DWI/ADC, T1/T2WI and DCE sequences. Clinical data and pathology reports were also retrieved from the system and reviewed for the study.

**Figure 1:**
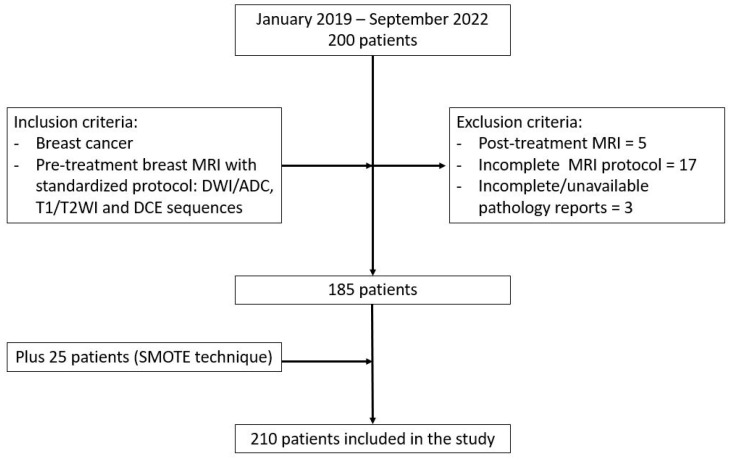
Study population.

Exclusion criteria were patients with inadequate or incomplete MR images and pathology and immunohistochemistry reports. A total of 185 patients were included in the study, together with an additional 25 patients from the SMOTE augmentation method (Figure 1).

### 2.2. Pathology and Immunohistochemistry Reports

All patients underwent a core-needle biopsy evaluation by pathologists using immunohistochemical (IHC) analyses. Pathology data were retrieved from the medical records, and tumor type (“no special type” (NST) or special types such as mucinous or medullary) and IHC characteristics ER, PR and HER2 status and ki67% proliferation index. ER and PR status were recognized as positive if at least 1% positive tumor nuclei were present in the sample, otherwise they were deemed negative. Ki67 expression level ≥20% was defined as high, otherwise it was defined as low. Human epidermal growth factor (HER2) status was considered positive if IHC showed 3+, otherwise the fluorescence in situ hybridization (FISH) test was used to check HER2/neu gene status.

### 2.3. MR Acquisition

All MRI examinations were performed using two 1.5 Tesla MRI machines (Siemens Magnetom Symphony TIM and Altea) with a dedicated 18-channel phased-array breast coil as a receiver.

The breast MRI protocol included five sequences. The DWI sequence was a 2D, single-shot, dual spin echo-planar sequence with standardized parameters of TR 4870 ms and minimum TE, with a flip angle of 90°. We used a 192 × 192 acquisition matrix for DWI sequence with a FOV of 38 cm, a slice thickness of 4 mm and a slice gap between 0 and 1 mm. The acquisition time was approximately 2 min for 5 b-values of 0, 200, 400, 600 and 800. We automatically calculated ADC-derived maps using the method provided by the vendor. All breast MRIs included a nonenhanced T1WI, T2WI sequence with a resolution of 0.6 mm × 0.6 mm, a slice thickness of 2 mm and TIRM sequence. Dynamic contrast-enhanced (DCE) images were obtained using a T1WI vibe fat sat dynamic sequence with a nonenhanced and five post-contrast phases after gadolinium administration. The DCE sequence parameters were as follows: TR = 4.66 ms, TE = 2.3 ms, slice thickness of 1.3 mm, with a gadolinium dose of 0.2 mL/kg and a debit of 3 mL/s.

### 2.4. Tumor Segmentation and Radiomic Feature Extraction

**Figure 2:**
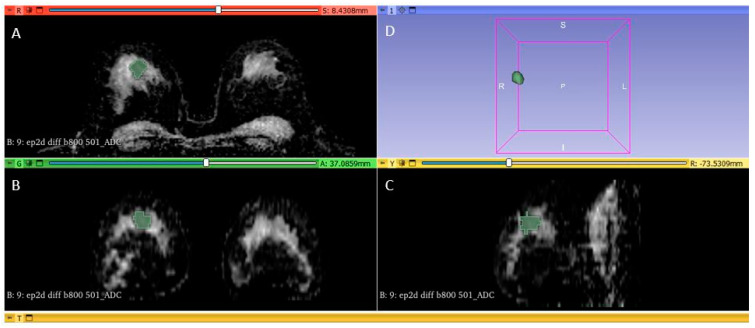
Breast tumor segmentation: A 34-year-old patient with ER/PR/HER2 negative (triple negative) breast cancer mass, segmented on axial (**A**), coronal (**B**) and sagittal (**C**) ADC images. The whole-tumor volume is highlighted in image (**D**).

Whole-tumor segmentation and radiomic feature extractions from ADC maps were performed using the publicly available 3D Slicer Software (version 4.10.2, available at: https://www.slicer.org/, accessed on 11 March 2023). One breast radiologist (M.S.) with more than 7 years of experience performed all the segmentations. The segmented area corresponded to the tumor drawn directly along the visible tumor margins on the ADC maps. DCE-MRI was used to confirm tumor localization in cases that were equivocal on DWI alone. Artifacts have always been excluded from segmentation and a distance of at least 2 mm from the metal clips marker was maintained. Radiomic analysis included the calculation of features derived from first-order histogram (Figure 2).

### 2.5. Statistical Analysis

Statistical analysis was conducted using MedCalc (version 19.2.6, Ostend, Belgium) and R software version 3.6.3. Univariate analysis using the Mann–Whitney U-test identified statistically significant differences in features between the ER/PR positive and ER/PR negative groups. Statistically significant features were further included in the multivariate analysis, using binary logistic regression (enter method) to identify independent predictors of ER/PR status and to build the radiomics model. We considered *p* values < 0.05 as statistically significant. Further, pathological and IHC data were added to the radiomics model in order to obtain a combined model. The ROC (receiver operating characteristic) curve with derived AUC (area under the curve), sensitivity, specificity and accuracy were used to evaluate the performance of individual radiomics features, radiomics score and combined radiomic model for the prediction of ER/PR status in both training and validation sets. Comparisons of the ROC curves were conducted using the DeLong method.

## 3. Results

### 3.1. Patient Characteristics

Synthetic Minority Oversampling Technique (SMOTE) was used to improve random oversampling and to augment the 185 patients with 25 more patients. A total of 210 patients (mean age 46.3) were included in the study. The patients were randomly divided into training (150 patients) and validation (60 patients) groups, in a 3:1 ratio. In the training group, 84 (56%) patients were ER/PR positive and 66 (44%) were ER/PR negative, while in the validation group, 29 (48%) patients were ER/PR negative and 31 (52%) were ER/PR positive. Patient characteristics, pathology and IHC data are summarized in Table 1. In the training group, pathology tumor type, histological grade and ki67% proliferation index values were significantly different between ER/PR negative and ER/PR positive patients, and this was confirmed in the validation dataset.

### 3.2. Feature Selection and Radiomics Score Construction: Training Set

A total of 18 radiomic features were extracted from the ADC images of each tumor/patient using MEdCalc. To develop the radiomics model, we started by performing univariate analyses of individual radiomic features between the ER/PR positive and ER/PR negative groups. We selected 14 features with *p*-value < 0.05 for the next step (Appendix A: Table A1).

We constructed the radiomics scores by including the 14 radiomics features and their coefficients in a multivariate logistic regression analysis (Appendix A: Table A2). We added ki67%, pathology tumor type and histological grade to the radiomics model in order to obtain a combined model.

**Figure 3:**
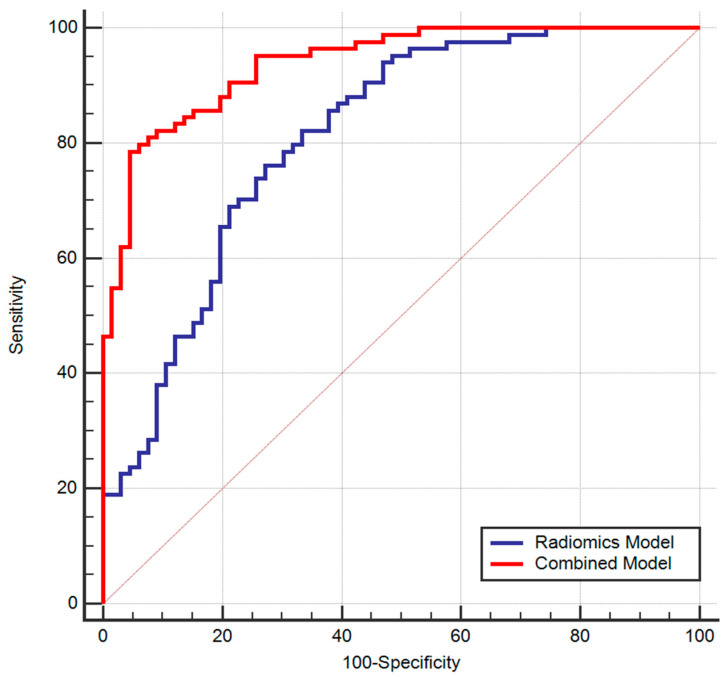
ROC curves of radiomics and combined models for predicting breast cancer molecular subtypes.

Individual radiomic features gave an AUC between 0.59–0.78, while the radiomics model reached an AUC of 0.81, with a sensitivity of 76% and a specificity of 72% (*p*-value < 0.0001) in differentiating ER/PR positive and negative tumors. The combined radiomics–pathologic model gave a higher AUC of 0.93, a sensitivity of 78% and a specificity of 95% (*p*-value < 0.0001), and the difference between ROC curves was statistically significant (*p*-value < 0.0001, 95% CI 0.066-0.18, Std error of 0.03), as shown in Figure 3. The diagnostic performance of all radiomics features and the radiomics and combined model are summarized in Table 2.

### 3.3. Testing the Radiomics and Combined Model: Validation Set

**Figure 4:**
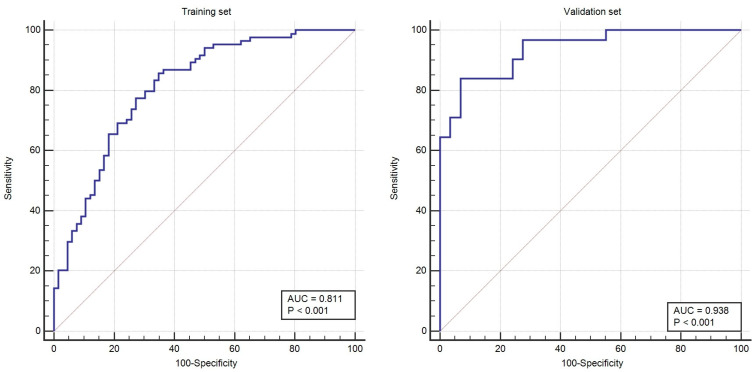
ROC curves of radiomics models of training and validation sets.

We repeated the multivariate analysis using the validation set (Appendix A: Table A2). The performance of the radiomics model in predicting ER/PR status in breast cancer patients was confirmed using the validation set, yielding an AUC of 0.93 (95% CI, 0.84–0.98, std error of 0.02) (Figure 4).

In the training group, we obtained a cut-off value of 0.56; however, the validation group reached a sensitivity of 83.87 (95% CI: 66.3–94.5), a specificity of 93.18 (95% CI: 77.2–99.2), PPV of 92.9 (95% CI: 76.5–99.1) and NPV of 84.4 (95% CI: 67.2–94.7).

The performance of the combined model at predicting ER/PR-positive breast cancer patients was higher in the validation set, yielding an AUC of 1.00 (95% CI: 0.94–1.00).

## 4. Discussion

The present study aimed to assess the value of ADC texture analysis in predicting the ER/PR status of breast cancer. We observed that radiomic features performed well at differentiating ER/PR positive from ER/PR negative tumors.

The differences in incidence, response to treatment, outcome and prognosis of different types of breast cancers make this a topic of increasing interest even today, more than 15 years after their intrinsic, genetic definition. Genetic tests are not widely available and still remain expensive, and IHC has a high percentage of variability, especially in reporting hormonal status (ER and PR), thus more and more studies are focusing on the role of imaging in predicting breast cancer molecular status.

Furthermore, radiomics of data extracted from breast ultrasound images showed promising results in differentiating ER/PR negative tumors from ER/PR positive tumors. The AUC reached 0.83, with a sensitivity of 69% and a specificity of 91.4% in predicting triple-negative breast cancer (ER/PR/HER2 negative) [17]. We obtained a higher accuracy (AUC 0.98), explained by the fact that we used MR images. It is well known that MR images have better resolution, thus more features can be extracted.

With the increasing use of breast MRIs, authors have focused more on predicting hormonal status from MR images. Studies analyzing DCE-MRI values such as Ktrans, Ve—volume of extravascular leakage space—or Kep—diffusion of contrast medium back to plasma)—reported contradictory results, with some authors reporting associations with ER/PR status and other authors failing to identify a surrogate marker for predicting hormone receptor status [18,19]. In addition, Ktrans and Kep are difficult and laborious to calculate and may require special processing software.

Radiomics based on DCE-MRI images reported highly variable AUCs (0.73–0.85) when predicting ER-positive status using a method that based on multiple intra-and peri-tumoral region-of-interest (ROI) [10]. The high variability in AUC values could be explained precisely by the use of ROI and not the entire tumor volume. Intratumoral heterogeneity is well known, with cystic, necrotic and hemorrhagic areas altering ADC data. While recent bleeding may contribute to lower values, cystic areas (necrotic or special tumors e.g., mucinous type) may have higher ADC values, even benign. Ideally, only solid, viable tumor parts should be included in the analysis, but here too are discussions (how large the ROI should be and where it should be placed) and the lack of standardization makes this almost impossible. Thus, analyzing the whole tumor is the best option that provides a more realistic value.

One unsupervised model based on DCE-MR was able to identify three novel breast cancer subtypes with distinct clinical outcomes and biological characteristics [11], but the fact that these were not perfectly matched with known molecular subtypes makes the study of limited clinical applicability. Furthermore, in terms of histology and IHC parameters, only ki67% was positively correlated with these novel subtypes.

However, the recent controversy about the safety of gadolinium-based contrast agents [16] has further prompted researchers to analyze the role of non-contrast sequences such as DWI and ADC.

Several studies assessed 2D ROIs on ADC maps to predict breast cancer molecular subtypes. HER2-positive tumors had higher ADC means compared with HER2-negative tumors, but with a modest AUC of only 0.605. The same authors reported no statistically significant differences between ER/PR positive and negative tumors in the quantitative histogram analysis based on ADC maps and in the qualitative visual assessment of DWI heterogeneity [20]. Our study observed a difference between negative and positive ER/PR, results that could be explained by the larger number of patients (165 versus 91) and the inclusion of the entire tumor volume (3D versus 2D). We chose not to qualitatively assess tumor heterogeneity on DWI precisely because of the high risk of non-reproducibility.

One study where tumors were segmented on DWI and segmentation ROIs were propagated onto ADC maps reported high accuracy in predicting hormonal status and some molecular types such as luminal B [21,22]. In addition, Leithner et al. [21] reported better results when first-order histogram parameters were obtained from ADC maps. However, due to the small number of patients in the above-mentioned study, the tumors were segmented on only one slice (with the largest tumor diameter) and the small number of patients prevented them from having a training group and a validation group.

In our study, the radiomics model was built on the training group and tested on an independent validation group. In addition, the breast tumors were segmented in 3D; thus, we obtained a whole-tumor volume from which we extracted radiomics data. The AUC of the radiomics model reached an AUC of 0.81 in the training cohort and was confirmed in the validation set, yielding an AUC of 0.92. We also tested a combined model based on radiomics data together with Ki67% proliferation index and histological grade and obtained a higher AUC of 0.93, which was also confirmed in the validation group. We chose not to analyze all five molecular subtypes, as a much larger number of patients is needed to obtain meaningful results. In addition, breast cancer treatment is guided by the presence or absence of hormone receptors (hormonal treatment) and there are no treatment guidelines focused strictly on the molecular subtypes of breast cancer. Their importance lies particularly in their impact on prognosis.

Other authors reported combined models using whole-tumor histogram (ADC_90_) with margins and enhancement in differentiating triple-negative breast cancers (ER/PR/HER2-negative) from other subtypes, with an AUC of 0.83 [23]. The lower AUC could be explained by the smaller study population or the addition of HER status to the analysis.

Consistent with our study, it was reported that the whole-volume apparent diffusion coefficient (ADC) histogram correlated with ki67% expression in breast masses [24]. However, the study focused on the benign–malignant differentiation of breast masses and not on different types of breast cancers, thus the study of ER and PR receptors was not possible. In addition, the ki67% proliferation index and histological grade do not have such a high variability compared with hormonal status (ER and PR); however, the former could still be used in combined models to further increase diagnostic accuracy.

There are limited studies that have included more than two molecular types of breast cancers in their analysis. Fan M. et al. extracted data from the tumor and the peritumoral stroma [14] and generated combined models from all regions. They achieved an overall AUC of 0.80 in the classification of four tumor subtypes: luminal A, luminal B, HER2 enriched and triple-negative basal-like.

Encouraging results have been reported in the use of other imaging methods in the diagnosis and characterization of breast tumors, suggesting the potential clinical value of mammography-based radiomics. However, in a recently published scoping review in which predicting breast cancer characteristics with radiomics is included, the final conclusion was that further efforts are required to standardize radiomics and select relevant mammographic radiomic features [25].

As regards contrast-enhanced spectral mammography (CESM), La Forgia et al. extracted histogram and texture parameters from 68 lesions on CESM and, using a multivariate linear discriminant analysis, found that for the tasks to differentiate ER status, PR status, Ki67, grade, TNBC status, and HER status, AUC values of 83.79%, 75.50%, 84.80%, 79.85%, 76.80%, and 90.89%, respectively, had to be achieved [26]. In another study conducted by Marino et al., tumor features extracted from CESM were classified using a machine learning classification method with the aim of characterizing breast lesions. The authors showed in their retrospective study of 103 breast cancer samples that radiomics analysis of CESM was able to differentiate invasive from non-invasive tumors and to define their hormone receptor status and tumor grades [27]. A recently published study by Dominique C et al. goes a step further and evaluates a deep learning model based on CESM to determine the receptor status and molecular type of breast cancer. The model was able to identify ER, but not PR, status. Furthermore, the models developed in this study seem to take their decision on the ring of the dual-energy subtracted images (DES) and the ill-defined or spiculated margins of the low-energy (LE) images even though the results obtained from the LE images were comparable to the full field digital mammography, while those obtained from the DES images were globally similar, with no obvious improvement in the results obtained by combining them with the majority voting system. Furthermore, the authors raise an interesting question regarding the need for contrast in selected patients (such as those with triple-negative tumors) [28].

It is well known that MRI has a higher sensitivity in detecting breast cancer compared with mammography, independent of tumor histology, tumor grading, single receptor status and molecular subtype [29]. However, there are no studies comparing radiomics data extracted from mammograms or MRIs.

The Androgen Receptor (AR) is emerging as an important factor in the pathogenesis of breast cancer (BC) and represents the latest addition to the worldwide IHC panel. AR is expressed in 70–90% of breast cancer cases, but its function seems to vary among different breast cancer subtypes [30]. The fact that AR inhibitors have recently been approved for the treatment of prostate cancer and could be a therapeutic tool for certain subgroups of breast cancer will increase the focus on their study (including radiomics).

One issue on the SMOTE analysis must be addressed. This synthetic minority over-sampling technique proposed by Chawla et al. [31] is a well-known over-sampling method employed in data pre-processing and has been used, with good results, in several studies [32,33,34]. Even if the CSC (cost-sensitive classifier) technique outperformed SMOTE, the latter proved that it can improve the predictive performance of models by solving imbalanced patient classification data [35]. We used SMOTE to synthetically augment our dataset with 50 more instances that were randomly distributed between the training and validation groups. The radiomics model achieved an AUC of 0.92 for the training cohort and correctly identified ER/PR status in the validation group (AUC of 1). A plausible explanation would be secondary to the SMOTE analysis and a higher incidence of synthetic data in the validation group, which may have, to some degree, homogenized the data; however, this hypothesis has not yet been tested.

Several research directions may result from the present study: (1) Studies comparing results obtained by radiomics-based MRI versus mammography versus ultrasound. (2) Inclusion of ARs (androgen receptors) in prediction models. (3) Cautious use of data augmentation methods and critical interpretation of results validated by larger population studies.

The current study has some limitations of note: (1) Although we had a larger study population compared with previously published studies, we still considered the group insufficient for analysis of all molecular subtypes. Thus, we opted only for differentiation based on hormonal status, which has major implications for treatment. (2) The study was unicentric. (3) All breast cancers were segmented manually (by M.S.), which might have introduced a certain level of observer-dependency and may not be feasible when analyzing large datasets; furthermore, because the segmentation was performed by a single radiologist, it was not possible to assess intra-and interobserver variability. (4) AR status was not available for all patients and therefore was not included in the current study.

## 5. Conclusions

Radiomic features extracted from ADC maps are able to predict the ER/PR status of breast cancer. Furthermore, a combined model (radiomics plus ki67% and tumoral grade) differentiated ER/PR positive tumors with greater accuracy. However, to confirm our results, both radiomics and combined models need to be externally validated in larger, multicentric studies.

## Figures and Tables

**Table 1:** Clinical, pathological and IHC characteristics of the study population.

Variable	Training Group			Validation Group		
	ER/PR − (*n* = 66)	ER/PR + (*n* = 84)	*p*-Value	ER/PR − (*n* = 29)	ER/PR + (*n* = 31)	*p*-Value
Age	44.87 ± 8.60	47.16 ± 8.48	0.09	44.37 ± 9.05	49.32 ± 8.88	0.03
Size	17.59 ± 4.39	17.25 ± 5.49	0.63	29.37 ± 15.56	14.41 ± 4.14	<0.001
Pathology			0.04			0.73
NST ^1^	64	69		27	27	
Other	2	15		2	4	
Ki67%	50.69 ± 25.89	26.16 ± 21.24	<0.001	60.89 ± 25.89	18.96 ± 18.05	<0.001
HER2			0.18			0.78
positive	2	8		4	4	
negative	64	76		25	27	
Histological grade			<0.001			<0.001
			
1	2	29		1	13	
2	4	37		4	15	
3	60	18		24	3	

^1^ NST = No special type, e.g., invasive carcinoma; other = invasive carcinoma with special features such as mucinous, medullar, papillary, lobular.

**Table 2:** Diagnostic performance of radiomics features, radiomics model and combined model at predicting ER/PR positive tumors from negative tumors.

Variable	Cut-Off Value	AUC (95% CI)	Sensitivity (95% CI)	Specificity (95% CI)	PPV (95% CI)	NPV (95% CI)
10th Percentile	≤764.4	0.716 (0.636–0.786)	60.71 (49.5–71.2)	74.24 (62.0–84.2)	75.0 (63.0–84.7)	59.8 (48.3–70.4)
90th Percentile	≤1389.8	0.763 (0.687–0.829)	89.29 (80.6–95.0)	54.55 (41.8–66.9)	71.4 (61.8–79.8)	80.0 (65.4–90.4)
Energy	≤94,304,540	0.634 (0.552–0.711)	83.33 (73.6–90.6)	39.39 (27.6–52.2)	63.6 (53.9–72.6)	65.0 (48.3–79.4)
Interquartile Range	≤264.25	0.639 (0.557–0.716)	73.81 (63.1–82.8)	60.61 (47.8–72.4)	70.5 (59.8–79.7)	64.5 (51.3–76.3)
Maximum	≤1377	0.731 (0.652–0.800)	57.14 (45.9–67.9)	83.33 (72.1–91.4)	81.4 (69.1–90.3)	60.4 (49.6–70.5)
Mean	≤1015	0.766 (0.690–0.832)	76.19 (65.7–84.8)	66.67 (54.0–77.8)	74.4 (63.9–83.2)	68.7 (55.9–79.8)
Mean Absolute Deviation	≤122.1356	0.667 (0.585–0.741)	50.00 (38.9–61.1)	78.79 (67.0–87.9)	75.0 (61.6–85.6)	55.3 (44.7–65.6)
Median	≤997	0.766 (0.690–0.832)	71.43 (60.5–80.8)	72.73 (60.4–83.0)	76.9 (66.0–85.7)	66.7 (54.6–77.3)
Range	≤874	0.665 (0.584–0.740)	60.71 (49.5–71.2)	69.70 (57.1–80.4)	71.8 (59.9–81.9)	58.2 (46.6–69.2)
Robust Mean Absolute Deviation	≤105.75	0.624 (0.541–0.702)	69.05 (58.0–78.7)	54.55 (41.8–66.9)	65.9 (55.0–75.7)	58.1 (44.8–70.5)
Root Mean Squared	≤1037.9215	0.780 (0.705–0.844)	77.38 (67.0–85.8)	71.21 (58.7–81.7)	77.4 (67.0–85.8)	71.2 (58.7–81.7)
Total Energy	≤502,487,200	0.649 (0.567–0.725)	46.43 (35.5–57.6)	80.30 (68.7–89.1)	75.0 (61.1–86.0)	54.1 (43.7–64)
Uniformity	>0.0455	0.596 (0.513–0.675)	89.29 (80.6–95.0)	28.79 (18.3–41.3)	61.5 (52.2–70.1)	67.9 (47.6–84.1)
Variance	≤42,112.37	0.677 (0.595–0.751)	70.24 (59.3–79.7)	59.09 (46.3–71.0)	68.6 (57.7–78.2)	60.9 (47.9–72.9)
Radiomics Model	>0.5699	0.811 (0.739–0.870)	76.19 (65.7–84.8)	72.73 (60.4–83.0)	78.0 67.5–86.4	70.6 (58.3–81.0)
Combined Model	>0.6631	0.938 (0.887–0.971)	78.57 (68.3–86.8)	95.45 (87.3–99.1)	95.7 (87.8–99.1)	77.8 (67.2–86.3)

## Data Availability

Data available on request due to privacy restrictions.

## Appendix A

**Table A1:** Univariate analysis of radiomic features.

Variable	Training Group			Validation Group		
	ER/PR − (*n* = 66)	ER/PR + (*n* = 84)	*p*-Value	ER/PR − (*n* = 29)	ER/PR + (*n* = 31)	*p*-Value
10th percentile	858.76 ± 152.69	703.54 ± 257.58	<0.001	751.01 ± 236.85	618.69 ± 298.62	0.06
90th Percentile	1404.14 ± 278.01	1147.33 ± 244.14	<0.001	1271.67 ± 265.68	1146.55 ± 259.51	0.07
Energy	154,079,091.87 ± 187,947,003.67	64,601,088.59 ± 70,687,481.87	0.005	383,070,294.27 ± 640,219,396.08	100,544,142.77 ± 241,933,338.28	0.02
Entropy	4.27 ± 0.65	4.03 ± 0.65	0.05	4.61 ± 0.60	3.96 ± 0.97	0.003
Interquartile Range	296.92 ± 122.50	242.24 ± 124.70	0.003	278.17 ± 142.01	279.01 ± 167.82	0.98
Kurtosis	3.50 ± 1.20	3.31 ± 1.23	0.17	3.66 ± 1.25	2.88 ± 0.86	0.007
Maximum	1698.28 ± 424.94	1390.76 ± 336.63	<0.001	1664.58 ± 361.44	1360.29 ± 322.37	0.001
Mean Absolute Deviation	182.88 ± 71.28	146.99 ± 67.02	<0.001	168.64 ± 71.25	173.44 ± 87.70	0.81
Mean	1109.55 ± 181.18	915.11 ± 228.80	<0.001	997.24 ± 230.44	873.56 ± 250.53	0.04
Median	1104.13 ± 191.24	902.88 ± 230.35	<0.001	974.86 ± 207.09	873.66 ± 260.92	0.11
Minimum	598.79 ± 236.58	541.33 ± 297.31	0.09	497.65 ± 320.07	472.00 ± 341.73	0.76
Range	1119.47 ± 527.31	849.42 ± 389.35	0.001	1166.93 ± 500.50	888.29 ± 455.84	0.02
Robust Mean Absolute Deviation	116.80 ± 46.24	100.41 ± 49.95	0.009	117.56 ± 58.57	116.58 ± 67.18	0.95
Root Mean Squared	1153.30 ± 196.44	938.48 ± 222.09	<0.001	1024.29 ± 217.98	908.51 ± 238.39	0.05
Skewness	0.028 ± 0.81	0.28 ± 0.56	0.06	0.46 ± 0.61	0.25 ± 0.62	0.19
Total Energy	2,405,590,407.93 ± 2,892,922,045.66	910,894,640.46 ± 839,674,754.21	0.002	6,238,029,521.44 ± 10,954,711,473.74	827,194,014.58 ± 1,298,673,230.16	0.008
Uniformity	0.067 ± 0.03	0.07 ± 0.04	0.04	0.05 ± 0.02	0.09 ± 0.07	0.006
Variance	66,273.47 ± 57,143.64	40,332.36 ± 38,935.60	<0.001	52,022.41 ± 47,979.07	56,528.80 ± 58,645.87	0.74

**Table A2:** Multivariate analysis of radiomic features.

Variable	Training Group			Validation Group		
	Coefficient	*p*-Value	Odds Ratio (95% CI)	Coefficient	*p*-Value	Odds Ratio (95% CI)
10th percentile	0.000	0.975	1.00 (0.99–1.00)	0.017	0.490	1.02 (0.96–1.06)
90th Percentile	−0.002	0.56	0.99 (0.99–1.00)	0.032	0.225	1.03 (0.98–1.08)
Energy	0.000	0.379	1.00 (1.00–1.00)	0.00	0.526	1.00 (1.00-1.00)
Interquartile Range	0.000	0.977	0.9998 (0.98–1.01)	0.005	0.869	1.005 (0.94–1.07)
Maximum	0.004	0.031	1.00 (1.00–1.00)	0.006	0.297	1.006 (0.99–1.02)
Mean Absolute Deviation	−0.019	0.118	0.98 (0.95–1.00)	0.144	0.266	1.15 (0.89–1.49)
Mean	0.002	0.567	1.00 (0.99–1.01)	−0.0002	0.998	0.99 (0.81–1.23)
Median	−0.002	0.493	0.99 (0.99–1.00)	0.072	0.023	1.07 (1.00–1.14)
Range	−0.001	0.308	0.99 (0.99–1.00)	−0.012	0.065	0.98 (0.97–1.00)
Robust Mean Absolute Deviation	0.026	0.094	1.02 (0.99–1.05)	−0.100	0.387	0.90 (0.71–1.13)
Root Mean Squared	−0.006	0.195	0.99 (0.98–1.00)	−0.136	0.177	0.98 (0.715–1.063)
Total Energy	0.000	0.039	1.00 (1.00–1.00)	0.00	0.440	1.00 (1.00–1.00)
Uniformity	−2.694	0.649	0.06(0.00–7446.19)	42.990	0.185	4.68 × 10^18^ (1.01509 × 10^−9^–21.59172 × 10^45^)
Variance	0.000	0.709	1.00 (1.00–1.00)	0.00001	0.849	1.00 (0.99–1.00)
Constant	4.454			−0.9538		
